# Functional connectivity of intrinsic cognitive networks during resting state and task performance in preadolescent children

**DOI:** 10.1371/journal.pone.0205690

**Published:** 2018-10-17

**Authors:** Ping Jiang, Virve Vuontela, Maksym Tokariev, Hai Lin, Eeva T. Aronen, YuanYe Ma, Synnöve Carlson

**Affiliations:** 1 Neuroscience Unit, Department of Physiology, Faculty of Medicine, University of Helsinki, Helsinki, Finland; 2 Department of Neuroscience and Biomedical Engineering, and Advanced Magnetic Imaging Centre, Aalto NeuroImaging, Aalto University School of Science, Espoo, Finland; 3 Huaxi Magnetic Resonance Research Center, Department of Radiology, West China Hospital of Sichuan University, Chengdu, China; 4 Child Psychiatry, Children’s Hospital, University of Helsinki and Helsinki University Hospital, Helsinki, Finland; 5 Pediatric Research Center, Laboratory of Developmental Psychopathology, University of Helsinki and Helsinki University Hospital, Helsinki, Finland; 6 Life Science and Technology, Kunming University of Science and Technology, Kunming, China; University of Texas at Austin, UNITED STATES

## Abstract

Earlier studies on adults have shown that functional connectivity (FC) of brain networks can vary depending on the brain state and cognitive challenge. Network connectivity has been investigated quite extensively in children in resting state, much less during tasks and is largely unexplored between these brain states. Here we used functional magnetic resonance imaging and independent component analysis to investigate the functional architecture of large-scale brain networks in 16 children (aged 7–11 years, 11 males) and 16 young adults (aged 22–29 years, 10 males) during resting state and visual working memory tasks. We identified the major neurocognitive intrinsic connectivity networks (ICNs) in both groups. Children had stronger FC than adults within the cingulo-opercular network in resting state, during task performance, and after controlling for performance differences. During tasks, children had stronger FC than adults also within the default mode (DMN) and right frontoparietal (rFPN) networks, and between the anterior DMN and the frontopolar network, whereas adults had stronger coupling between the anterior DMN and rFPN. Furthermore, children compared to adults modulated the FC strength regarding the rFPN differently between the brain states. The FC within the anterior DMN correlated with age and performance in children so that the younger they were, the stronger was the FC, and the stronger the FC within this network, the slower they performed the tasks. The group differences in the network connectivity reported here, and the observed correlations with task performance, provide insight into the normative development of the preadolescent brain and link maturation of functional connectivity with improving cognitive performance.

## Introduction

Cognitive control, the ability to execute voluntary, goal-directed behavior, requires collaboration of core regions of several brain networks [1–4]. This ability continues to improve from childhood to adulthood as reflected by an age-related gradual increase in response speed and accuracy in the performance of inhibitory control [5] and working memory (WM) [6–8] tasks. The development of cognitive control is related to the maturation of the core regions of the executive system [9–10], and to increased integration among widely distributed brain circuitries [11–12]. The relationship between the maturation of brain networks and age-related improvement in children’s cognitive abilities is still not well understood.

Functional connectivity (FC) refers to temporal correlations of neuronal activation patterns in different brain regions [13] and can be measured with functional magnetic resonance imaging (fMRI). Brain areas that during “resting state” show spontaneous, temporally correlated low-frequency fluctuations of the blood oxygen level dependent (BOLD) signal, form functional networks. Modern brain imaging techniques, and data driven analysis methods, such as independent component analysis (ICA) [14] allow to investigate brain network connectivity not only during resting state but also during task performance. In order to better understand the normative development of brain networks that support cognitive functions, and their role in cognitive task performance, we identified several intrinsic connectivity networks (ICNs) using resting state fMRI in 7-11-year-old children and young adults. We then compared, between the groups, the within- and the between-network connectivity of these networks in resting state, during task performance and between these brain states. The within-network connectivity refers to the strength of correlations between the time courses within the network, and the between-network connectivity, to the strength of correlations between the networks.

Functional MRI studies have revealed a number of ICNs during resting state [15–16], which reflect the intrinsic functional architecture of the brain [17]. The major representative ICNs include low-level unimodal processing networks relevant to vision, audition, and actions, and neurocognitive networks related to high-level cognitive processing. In neuroimaging literature, neurocognitive networks have been variably named either after the functional characteristics of the networks or according to the core brain regions comprising them. We focused on the default mode (DMN), dorsal attentional (DAN), frontoparietal (FPN), cingulo-opercular (CON) and frontopolar networks [15, 18–21]. The DMN is suggested to play an important role in cognitive control through effective allocation of attentional resources to intrinsic thought or extrinsic stimuli [22–27], whereas other neurocognitive networks, including the FPN, DAN, CON, and the frontopolar network, are involved e.g. in attention, memory and executive functions [16,19,24,26, 28–29].

Recent neuroimaging studies in adults have shown that the ICN architecture is highly consistent during rest and cognitive tasks [17,21]. The close correspondence between intrinsic and task-evoked connectivity implies, in line with the Hebbian theory [30], that the spontaneous activity may represent a history of repeated co-activations between brain regions during tasks [31–32]. Although adults have a stable ICN architecture, the FC within and between the networks undergoes dynamic changes during task performance [33]. In general, the execution of cognitive tasks breaks the baseline network connectivity and creates a task-specific pattern of FC [34], i.e. the within-network connectivity decreases and between-network connectivity increases [17]. A recent neuroimaging study in adults reported that, compared to the resting state, cognitive task performance decreased FC within the DMN and increased integration between the DMN and brain regions of the CON [35]. These FC changes correlated with the performance of the tasks: the greater the FC change the shorter the response time and the better the accuracy. Another recent study in adults, using magnetoencephalography, graph theory and WM tasks, demonstrated that greater cognitive demands were associated with less clustered and less modular brain network organization, leading to more efficient information processing [36].

Developmental neuroimaging studies have reported robust large-scale ICNs in early [37] and late [38] childhood. The visual, auditory, and sensorimotor networks are detectable in infants [39], and the major nodes of the DMN are functionally connected in 2-year-olds [40], however, the architecture of the DMN continues to develop during childhood [41–42]. Previous studies using resting state fMRI have suggested that the large-scale network organization is established in adolescence [12, 43–44], but the fine-tuning of FC continues during development, including changes in within- and between-network connectivity [12,31, 45–49]. These functional changes parallel structural modifications that occur during development, such as regional alterations in gray matter volume [50–51] including synaptic and dendritic proliferation and pruning [52], and greater myelination of the cortex [52–55]. Concurrently, cognitive abilities including WM, attentional control and suppression of distraction, improve through childhood to young adulthood [5–8,56]. Neuropsychiatric studies have reported that, compared to controls, attenuated deactivation of the DMN during task performance is associated with lapses of attention in children with attention deficit hyperactivity disorder [57], and decreased FC between the right intraparietal sulcus and dorsal frontal regions is coupled with lower accuracy in WM tasks in girls with Turner syndrome [58]. Currently, there is a gap in understanding how brain network FC differs between resting state and task performance in typically developing populations.

The growing understanding of the neurodevelopment of the structure and function of the brain, and of the brain network FC, has led to models explaining the normative functional development of the brain. Brain activation patterns during cognitive task performance have been shown to change over the development so that the relatively diffuse cortical activation observed in children becomes more focal over development [59]. Fair and colleagues [31], on the other hand, put forward a model suggesting a local-to-distal shift in FC of networks across maturation indicating that children, compared to adults, have stronger short-distance connections, and that over the development the FC strength between distant nodes increases.

Here, we used fMRI during resting state and visual WM tasks to investigate differences in FC in resting state, during task performance and between these two states in preadolescent 7-11-year-old children and young adults. We employed ICA, dual regression and permutation tests to explore the architecture of ICNs and age-related differences in resting state and task-evoked FC. Based on earlier studies, reviewed above, suggesting that the organization of resting state networks is largely established in young, school-aged children, but the fine-tuning of FC continues, and on studies in adults indicating that the network connectivity during cognitive task performance compared to resting state changes so that the within-network connectivity decreases and between-network connectivity increases, we tested the following hypotheses: 1) Although the general architecture of the ICNs may already be established in 7-11-year-old children, the FC within and between the ICNs during resting state differs from that in adults. 2) According to the model that indicates a local-to-distal shift in FC across maturation [31], we predicted that, during tasks, children compared to adults have stronger within-network FC and weaker between-network FC. 3) We also predicted that, in both groups, task performance compared to the resting state alters the FC within and between the ICNs. We anticipated that adults exhibit weaker within- and stronger between-network connectivity during tasks compared to resting state, whereas these changes would be less pronounced in children.

Although literature is accumulating about the FC of brain networks during resting state and, to a lesser extent, during task performance, there is a need to better understand the neural mechanisms that enable transitions between different brain states, and how these mechanisms develop from childhood to adulthood.

## Materials and methods

### Participants

A total of 16 children (aged 7–11 years, mean age 9.1 ± 1.4 years, 11 males) and 16 young adults (aged 22–29 years, mean age 25.2 ± 2.3 years, 10 males) with no prior neurological or psychiatric diseases participated in this study. The Ethics Committee for Pediatrics, Adolescent Medicine and Psychiatry at the Helsinki University Central Hospital approved the study, and all children and their guardians and all adults provided written informed consents prior to participation in accordance with the Declaration of Helsinki.

### Image acquisition and scanning procedure

Scanning was performed at the Advanced Magnetic Imaging Centre of Aalto University first using General Electric (GE) Signa (Milwaukee, WI, USA) (16 children and 11 adults) 3 T MRI scanner with a standard 8-channel head coil and then, due to an update of the scanner, using Siemens MAGNETOM Skyra (Erlangen, Germany) (5 adults) 3 T scanner with a 30-channel head coil. Functional images were obtained first during WM task performance and then during resting state using an identical gradient-echo planar imaging sequence (TR 2500 ms, TE 30 ms, flip angle 75°, FOV 220 mm, matrix size 64 x 64, in plane resolution 3.5 x 3.5 mm) for both scanners. The number of functional volumes in one task run was 184, resulting in a total of 552 volumes from the three task runs per each participant. The resting state run produced altogether 144 volumes. Each functional volume consisted of 43 (GE) or 45 (Siemens) axial slices of 3.5 mm with no inter-slice gap and covered the whole cerebrum and cerebellum. High-resolution anatomical T1-weighted MRI images were acquired using a spoiled-gradient-echo sequence (170 slices, FOV 256 mm, matrix size 256 x 256, voxel size 1.0 mm x 1.0 mm x 1.0 mm) with the GE scanner and magnetization-prepared rapid gradient-echo sequence (176 slices, FOV 256 mm, matrix size 256 x 256, voxel size 1.0 mm x 1.0 mm x 1.0 mm) with the Siemens scanner.

In the block-design visual 1-back WM tasks, a set of grey-scale images representing neutral faces and natural scenes were used as stimuli [60]. The participants performed four different types of visual 1-back tasks: two simple and two complex tasks. The simple 1-back tasks used only face (F task) or scene (S task) images as stimuli. The participants were instructed to press a button whenever the face in the F task or scene in the S task was the same as the previously presented image (duration of images 300 ms, inter stimulus interval (ISI) 1450 ms). The complex tasks (Sf and Fs tasks) required suppression of a task-irrelevant distractor between two targets (a face image in the Sf task and a scene image in the Fs task). The participants were instructed to attend to the target images and to ignore the distractors in between the two targets (duration of each image 300 ms, ISI 575 ms, inter target interval (ITI) 1450 ms) and press a button whenever the target was the same as the one in the previous trial. In addition to the 1-back tasks, there was a rest condition (R) with visual fixation on a central cross on the screen when no task was performed. Each participant performed three separate runs in total, each of which contained two blocks of S, F, Sf, Fs and R conditions in a semi counterbalanced order. Each block included 20 trials of the task condition, thus, 120 trials of each task condition (S, F, Sf, Fs) were performed resulting in a total of 480 trials. The duration of the whole imaging session was approximately 40 min including a 6-min resting state dataset that was collected at the end of the imaging session. During the resting state, the participants were instructed to lay still with their eyes closed, think of nothing in particular and not to fall asleep. After the imaging session, the participants were interviewed by the investigators and asked about the course of scanning. They also filled in a questionnaire designed to evaluate the level of alertness during the first, middle and last parts of the scanning using a 3-point scale (1 = alert, 2 = tired, 3 = sleepy) and task difficulty using a 5-point scale (1 = very easy, 2 = easy, 3 = not easy, not difficult, 4 = difficult, 5 = very difficult), and reported if something, e.g. distress or discomfort, had affected the course of scanning. A two-way repeated-measures ANOVA showed that there were no significant differences in the reported alertness during scanning between the groups (see S1 File). Details about the scanning procedure were described in the work of Jiang and colleagues [60].

### Functional MRI data analysis

The resting state fMRI data were obtained in 14 of 16 children and all adults. Since recent studies [61–62] indicate greater inter-personal than scanner-related variability in the fMRI data and suggest that the data are reproducible and highly reliable across different scanners, we pooled the data from the two scanners for the analyses. However, we also investigated the independence of the results from the scanner by conducting the main FC analyses separately for each scanner’s data (see S1 File). Preprocessing of individual data consisted of brain extraction, motion correction, spatial smoothing (5 mm FWHM Gaussian kernel), and high-pass temporal filtering equivalent to 100 s (0.01 Hz). The global signal was not regressed out. Functional MRI data were registered to the individual's structural scan and the MNI152 standard space template [63] with a 2 mm resolution using FMRIB's Linear Image Registration Tool (FLIRT). Previous studies testing the usefulness of analyzing pediatric and adult neuroimaging data in a common stereotactic space [64–65] have shown that although there are small anatomical differences between adults and children older than seven years of age, these differences do not translate to spurious results in functional imaging data analyses. Thus, in order to make direct statistical comparisons of functional data between the two age groups, we used the common stereotactic space for the spatial normalization in children and adults. The task fMRI data were obtained from all subjects. The preprocessing of task fMRI data was similar as for the resting state data. One run of task fMRI data from three children was excluded from further analysis due to excessive head movement (> 3.5 mm, mean absolute displacement) during scanning.

In addition to the standard fMRI preprocessing, we used the FMRIB's ICA-based Xnoiseifier—FIX (v1.061 beta) [66–67] to clean-up the data in order to largely control the influences of head motion and other nuisance noise (e.g., cardiac pulse, respiration) on the results. The subject-level ICA-based artifact removal underwent four steps. First, all individual resting state and task fMRI data were analysed with ICA using Multivariate Exploratory Linear Optimized Decomposition into Independent Components (MELODIC) package in FSL [68] by probabilistic independent component analysis (PICA) [69]. The number of independent components (ICs) was automatically estimated using the Laplace approximation as implemented in MELODIC [69]. Second, to create a training dataset for FIX, one of the authors (P.J.) manually labeled the components into ‘signal’ and ‘noise’ from a sample of our datasets based on both the spatial and temporal characteristics. Hereafter, the leave-one-out (LOO) approach was used to evaluate the accuracy of the hand-classified data. The results provided an overall high accuracy with mean true-positive rates at 97.1% and 97.0% and true-negative rates at 90.3% and 90.4% for resting state and task data, respectively. Third, with the aid of the training dataset, FIX automatically classified single-session ICA output into 'good' and 'bad' components in the remaining resting state and task data. Finally, the bad components and motion confounds with 24 motion parameters were regressed out from the preprocessed 4D fMRI data to obtain the cleaned datasets. The details of the classification of ICA output can be found in S2 File. For the subsequent analysis, the three runs of cleaned task fMRI data in each subject were averaged into one session for further analyses.

For group ICA, the cleaned individual data of resting state and tasks were fed into the MELODIC for group-level decomposition by temporal concatenation approach and tensor-PICA analysis [70], respectively. The group ICA produced 52 components for resting state and 30 components for tasks. By visual inspection, an IC was categorized as an artifact when it had such characteristics as: 1) low spatial overlap with gray matter or high spatial overlap with the sagittal sinus, white matter, cerebrospinal fluid or brain’s boundary in structural templates, 2) a large number of small clusters, 3) predominantly high-frequency (> 0.1Hz) power in the time-course spectrum, and 4) the time series was bimodal or had sharp peaks or large jumps [67, 71]. Moreover, the component was not categorized as signal if it was driven by a single outlier subject or run [72]. Out of all the signal components, we identified the neurocognitive ICNs including the DMN, FPN, DAN, CON and the frontopolar network for further analyses. The naming of the networks in the current study was based on previous reports [18, 32, 73] and on the anatomical locations of the core brain regions of the networks. The neurocognitive ICNs obtained from the resting state data of 14 adults and 14 children were used as spatial templates for the following analyses, such as FSL’s dual regression approach to generate subject-specific spatial maps and time courses for each component [74]. The neurocognitive ICNs were also obtained from separate within-group ICA of the resting state data to represent the network architectures in each group.

### Between-group analyses and interactions between group and brain state

The between-group analysis of the resting state and task fMRI data was carried out using dual regression and permutation tests [74–75] that allow voxel-wise comparisons of FC patterns. Dual regression was used to generate subject-specific versions of the spatial maps and associated time-series. The dual regression procedure was carried out as follows. First, the combined group ICA spatial maps were obtained from the resting state and used as spatial regressors in a multiple regression analysis against the cleaned individual dataset. This resulted in a set of subject-specific time-series associated with each group-level spatial map. Then, the individual time-series data were demeaned, variance-normalized, and used as temporal regressors in a multiple regression analysis against the same dataset, resulting in a set of subject-specific spatial maps. Finally, we tested for statistically significant group differences of within-network connectivity using FSL's randomise nonparametric permutation-testing tool (5000 permutations) [76–77] with a threshold-free cluster enhancement (TFCE) method [78] to control for voxel-wise multiple comparisons across the whole brain. For multiple comparisons correction across the studied components, the resulting spatial maps were thresholded at a p-level of 0.05 using FDR correction [79].

Between-networks FC was examined with the FSLNets toolbox (http://fsl.fmrib.ox.ac.uk/fsl/fslwiki/FSLNets) that used the subject-specific time courses of each spatial map from the dual regression analysis to generate a 10 × 10 matrix of between-networks connection strengths for each subject. The correlation matrix was estimated by both full and partial correlations. The group comparisons of between-networks connectivity strengths were conducted separately for resting state and task data by permutation tests with multiple comparison correction [71].

We used a two-way repeated measures ANOVA in SPSS software (http://www-01.ibm.com/software/analytics/spss/) to examine whether the FC in the brain states (resting state and task performance) differed between the groups and whether there was a group x brain state interaction. In each subject, the strength of within-network FC was calculated as the average z-score derived from dual regression across all voxels in each of the neurocognitive networks. The strength of between-network FC was represented by the transformed z value from partial correlation analyses in FSLnets of connection strengths between networks during resting state and tasks.

**Fig 1:**
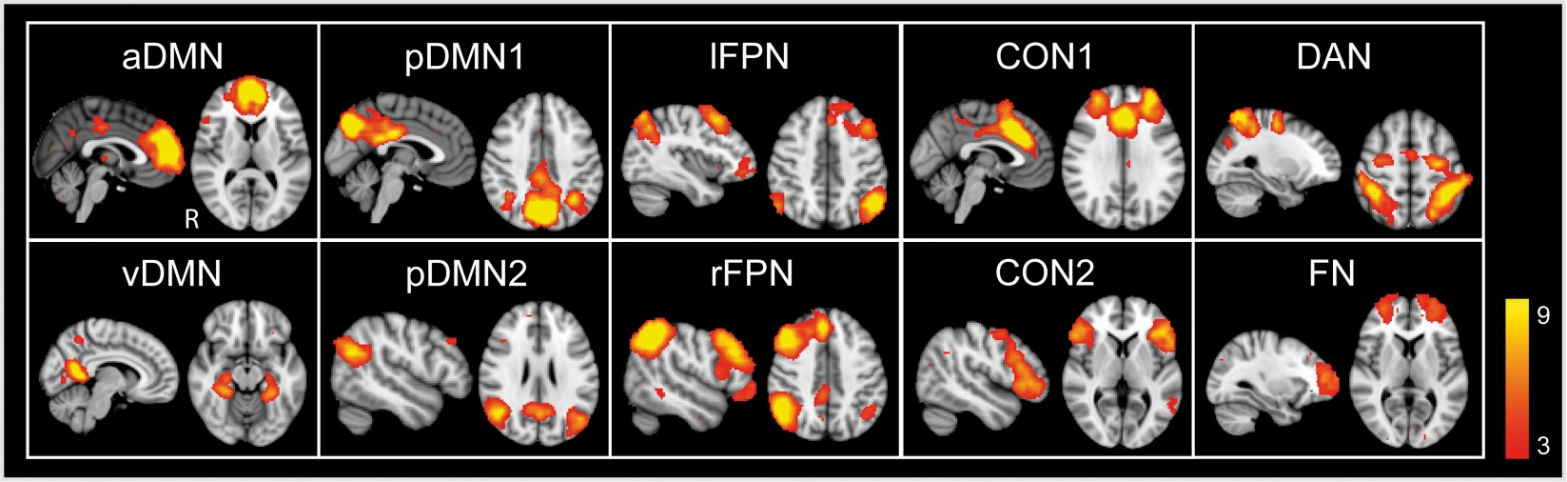
Neurocognitive intrinsic connectivity networks. The spatial maps of neurocognitive networks (thresholded at Z > 3) shown here represent the anterior, posterior and ventral subnetworks of the default mode network (a/p/v DMN), and the left and right frontoparietal (l/r FPN), dorsal attentional (DAN), cingulo-opercular (CON), and frontopolar (FN) networks. The spatial maps are displayed in sagittal and axial views and superimposed on the MNI152 standard space template image.

**Fig 2:**
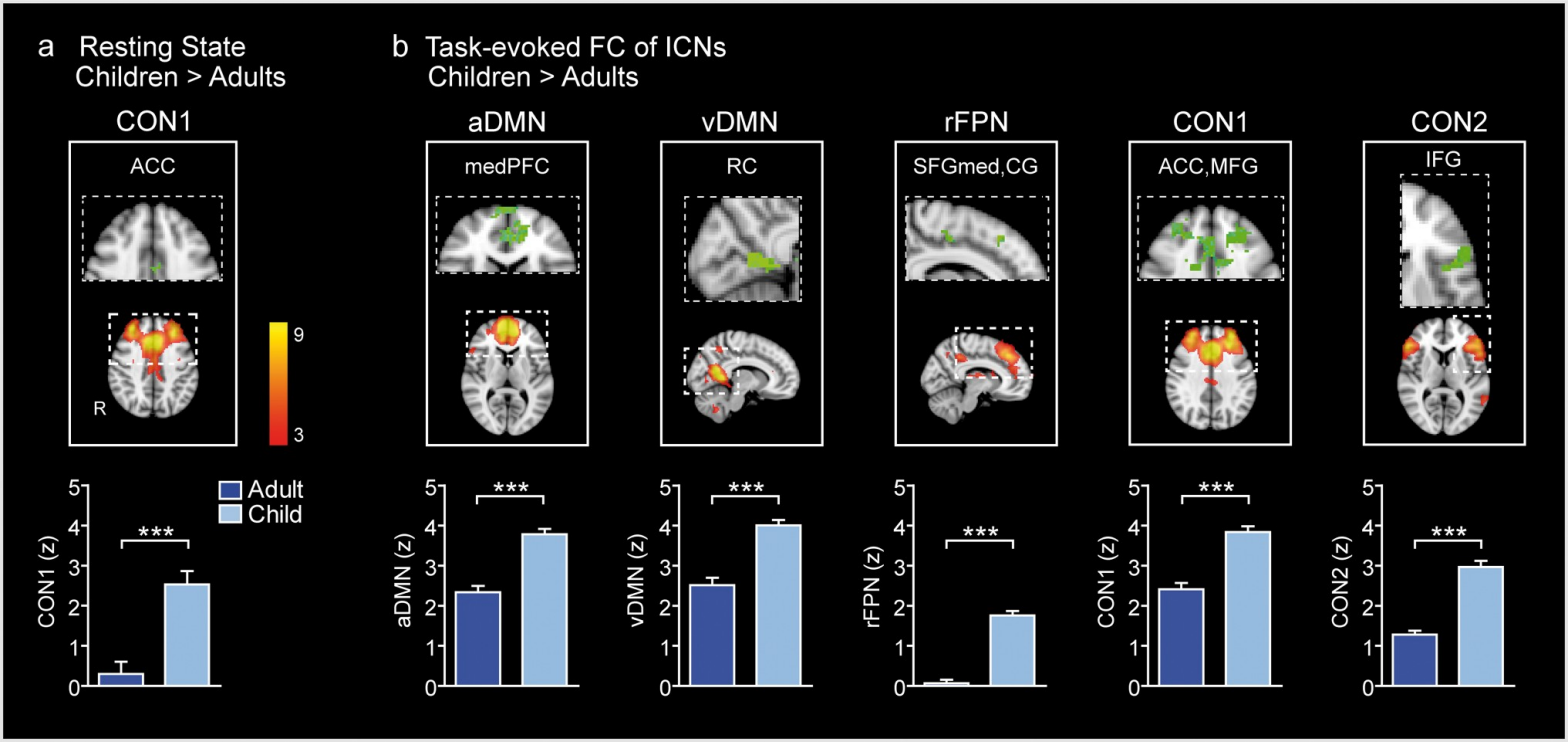
Group differences in within-network FC of the ICNs during resting state and tasks. Using combined group ICNs as templates for dual regression, the between groups comparison showed that (a) during resting state, children compared to adults, had a significantly stronger FC in the ACC within the CON1. (b) During tasks, children compared to adults had stronger FC in five ICNs representing the aDMN, vDMN, rFPN, CON1 and CON2. All comparison analyses were FDR corrected at p < 0.05 with a cluster size of > 10 contiguous voxels. The statistical maps of significant FC differences are presented in green, and the corresponding group ICN templates are shown in red/yellow. All statistical maps are displayed on selected slice planes of the MNI152 standard brain template. The columns illustrate the mean FC within the area that differed significantly between the groups. ACC, anterior cingulate cortex; CG, cingulate gyrus; CON, cingulo-opercular network; DMN, default mode network; FPN, frontoparietal network; IFG, inferior frontal gyrus; MFG, middle frontal gyrus; PFC, prefrontal cortex; RC, retrosplenial cortex; SFG, superior frontal gyrus; a, anterior; v, ventral; med, medial part; R, r, right; *** p < 0.001 (unpaired t-test).

**Fig 3:**
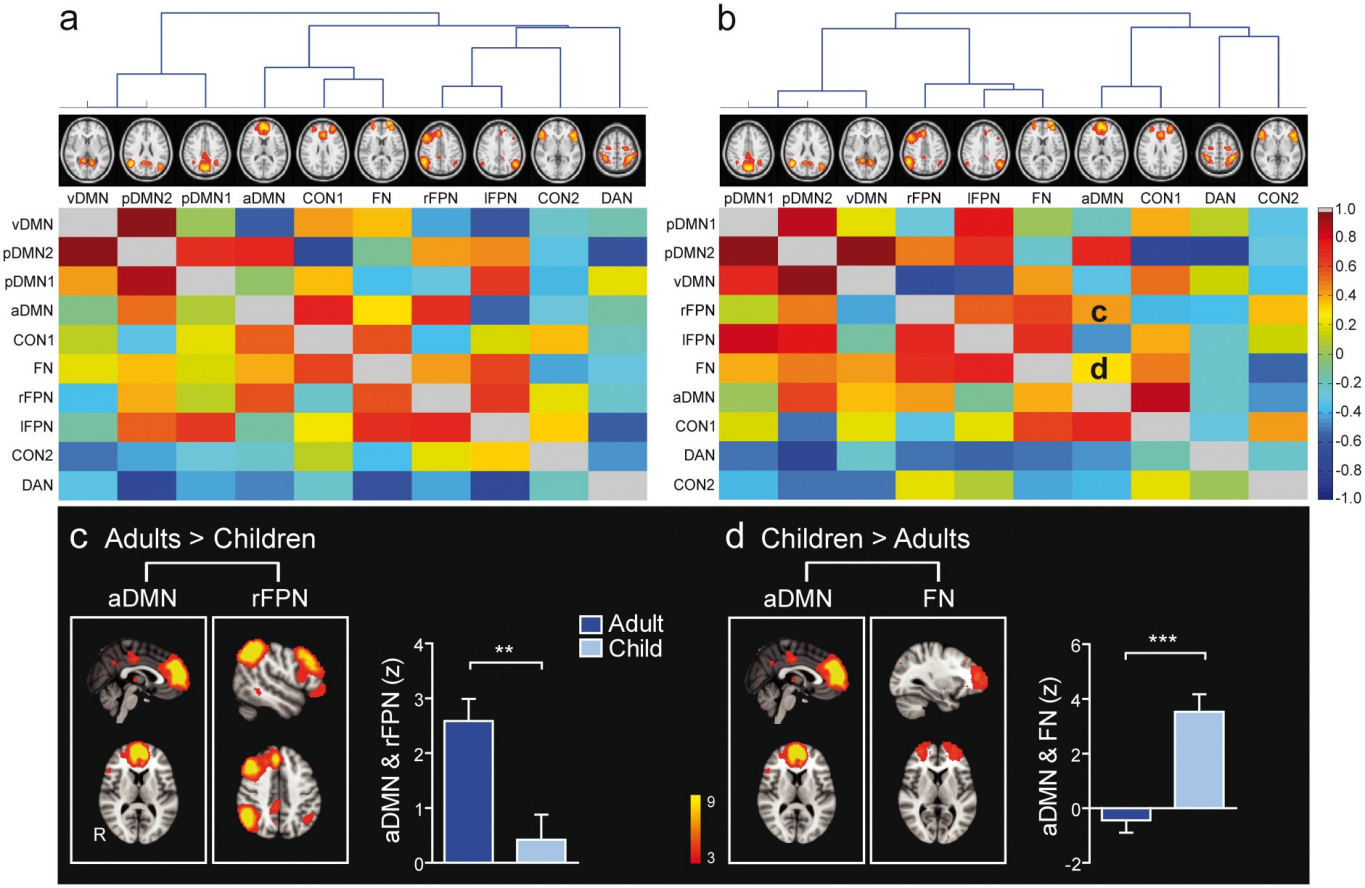
Hierarchical clustering, full and partial correlations and group differences in between-network FC of the ICNs. Hierarchical clustering of the neurocognitive networks of the combined group data during (a) resting state and (b) task performance. Full and partial correlations between the ICNs representing neurocognitive networks are displayed below and above the main diagonal, respectively. (c) Adults compared to children showed stronger connectivity between the aDMN and rFPN during tasks (indicated by letter c in panel b). (d) Children compared to adults had stronger connectivity between the aDMN and the frontopolar network during tasks (indicated by letter d in panel b). The significance level of all comparison analyses was multiple comparison corrected at p < 0.05 with cluster size > 10 contiguous voxels. The columns in c and d illustrate the mean FC between the networks that differed significantly between the groups. CON, cingulo-opercular network; DAN, dorsal attentional network; DMN, default mode network; FN, frontopolar network; FPN, frontoparietal network; a, anterior; p, posterior; v, ventral; l, left; R, r, right; ** p < 0.01, *** p < 0.001 (unpaired t-test).

**Fig 4:**
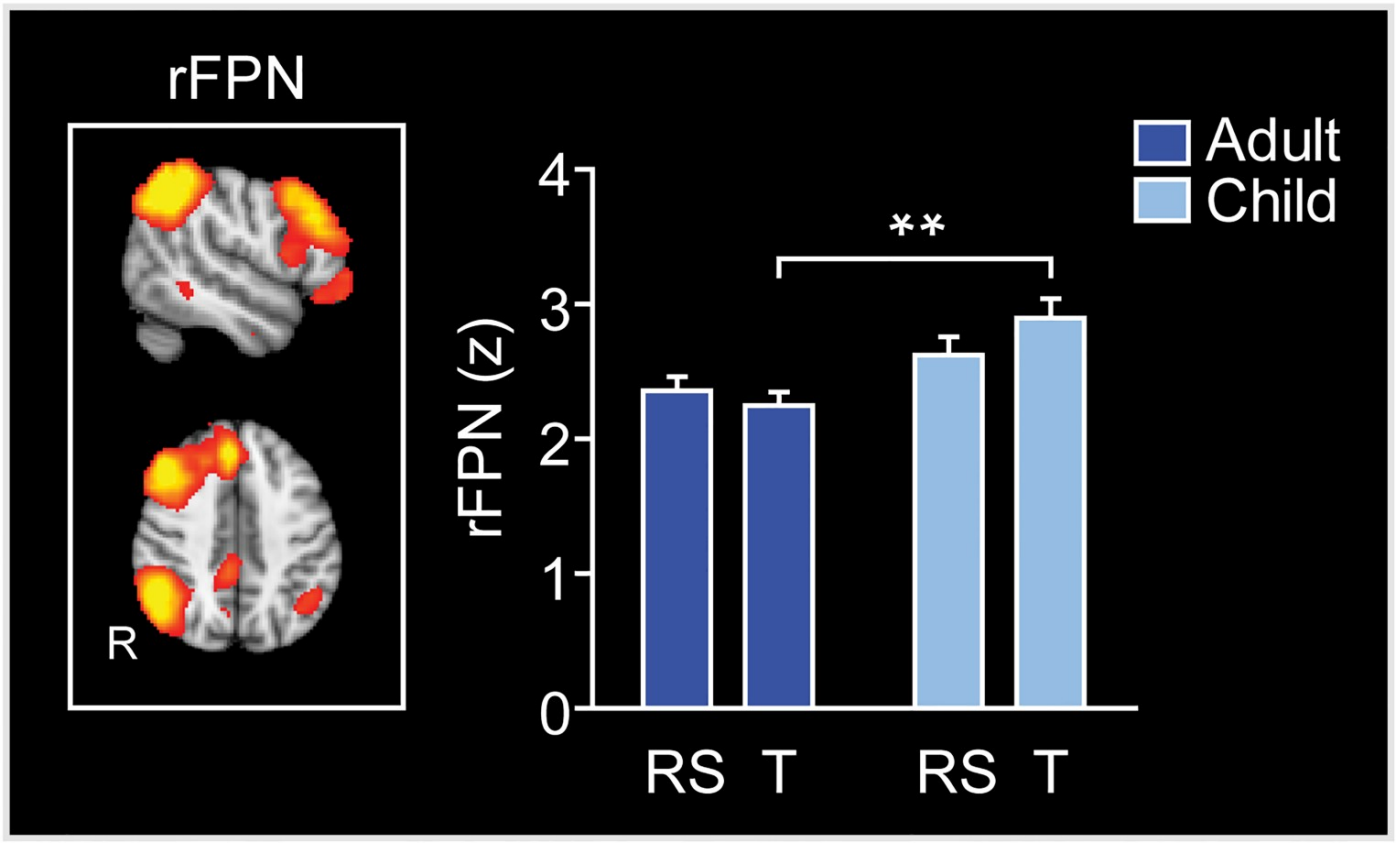
Group difference in FC strength of the rFPN between resting state and tasks. The rFPN showed significant group x brain state interaction of FC in the repeated measures ANOVA. The mean FC within the rFPN was significantly stronger in children than adults during tasks. The columns illustrate the average z-score across voxels in the rFPN. FPN, frontoparietal network; RS, resting state; T, tasks; R, r, right; ** p < 0.01 (two-way repeated measures ANOVA, followed by unpaired t-tests).

In the following, we present the pipeline of the fMRI data analysis:

1. FSL’s FIX was used to denoise the preprocessed individual fMRI data for further ICA of resting state and task data. The denoised fMRI data were used for the following group-level FC analyses.
2. The group-level decomposition of resting-state and task data into ICNs was performed using FSL’s MELODIC.
3. For resting state, the neurocognitive ICNs were obtained by combining the data from adults and children (Results, Fig 1). These data were used as spatial templates for dual-regression analyses on both resting state and task data to generate subject-specific versions of the spatial maps and associated time-series for each ICN. In addition, within-group ICA was performed separately for adults and children to illustrate the resting state network architecture within each group (S1 Fig).
4. Full and partial correlations of FC strengths between networks and their hierarchical clustering during resting state and tasks were generated by the FSLNets toolbox.
5. Group differences in resting state and tasks were analyzed using FSL’s Randomise tool. The input for the within- (Results, Fig 2) and between-network (Results, Fig 3) analyses were the subject-specific time-series from dual regression.
6. Individual mean z-scores of each ICN derived by dual regression representing within-network FC strength, and the transformed z-scores calculated by partial correlation analyses in FSLnets representing between-network FC strength, were used for the repeated measures ANOVA to examine brain state × group interaction of within- and between-network FC, respectively (Results, Fig 4).

### Behavioral data analysis

The behavioral data consisted of response times (RTs), number of hits, misses and false alarms (FA). The performance accuracy was calculated as sensitivity (d’), which represented differences between the inverse of the standard normal cumulative distribution of hits and FA [80]. Statistical analysis of behavioral parameters including RTs and d’ was conducted using SPSS software. In the tasks, the subjects responded by pressing a button only in the match conditions. Therefore, the amount of behavioral data collected during one type of WM task was relatively low. To increase statistical power of the correlation analyses, and because all four tasks measured visual 1-back WM performance, the behavioral data were collapsed across the four task conditions. We performed correlation analyses to investigate the relationships between the task-related FC of the ICNs and 1) age, and 2) behavioral performance (mean d’ and RTs). In each subject, the mean connectivity strength of the within-network FC was calculated as the average z-score derived from dual regression across all voxels in each of the networks and the strength of between-network FC was represented by the transferred z value of partial correlation analysis in FSLNets. Cook’s Distance [81] was calculated to identify possible outliers which were then excluded from the correlation analyses. The Bonferroni multiple comparison correction was performed for the number of statistical tests and the significance level was set at *p* < 0.017.

### Assessing effects of motion correction and motion residuals

After using FIX to clean the data, the level of motion-related noise was significantly reduced in adults’ and children’s data during resting state (comparison between the mean absolute displacement before and after FIX in each group, adults, *p* < 0.001; children, *p* = 0.0013) and tasks (adults, *p* < 0.001; children, *p* < 0.001) (Table A in S5 File), although it was still larger in children compared to adults. In addition, after using FIX, we compared the motion residuals of the resting state data to that of the task data in each group separately. The motion residuals of the resting state data and task data did not differ significantly from each other either in adults (*p* = 0.216) or children (*p* = 0.450).

To evaluate the impact of motion residuals on the group differences of FC during resting state and tasks, we calculated the voxel-wise Pearson’s correlation between the motion residuals and within-network FC metrics across participants separately in adults and children [82]. The motion residuals were indexed by the temporal mean voxel-wise metrics of the framewise displacement (mean FDvox), which was calculated by the toolbox DPABI (http://rfmri.org/dpabi). The FC metrics for each subject’s networks were represented by the z-score of the within-network FC derived from dual regression. The areas showing significant group differences of FC during resting state and tasks were used as masks for the correlation analyses. The FDR correction was used to control for voxel-wise multiple comparisons across the brain areas and the significance level was set at *p* < 0.05.

We also examined, using correlation analyses, whether any of the motion correction parameters (mean absolute displacement, maximum absolute displacement, mean relative displacement, maximum relative displacement) were associated with subjects’ age, RT, number of hits, misses, FAs or performance accuracy (d’).

## Results

### Neurocognitive networks from the combined group ICA of the resting state data

After visual inspection of the combined group ICA (see section Functional MRI data analysis, Pipeline point 3), 11 ICs were identified to represent the neurocognitive networks [15, 21, 83], including the DMN, FPN, DAN, CON and the frontopolar network (Fig 1). The DMN consisted of anterior (aDMN), posterior (pDMN) and ventral (vDMN) networks. The pDMN was further divided into two subnetworks (pDMN1, pDMN2). The core region of aDMN was located in the medial prefrontal cortex (medPFC), the pDMN had regions in the precuneus, posterior cingulate and angular gyruses, whereas the vDMN included regions in the precuneus, retrosplenial cortex, hippocampus, parahippocampal gyrus and occipital cortex. The FPN that consisted of right (rFPN) and left (lFPN) networks, included regions in the superior (SFG), middle (MFG), and inferior (IFG) frontal gyruses, and inferior parietal lobule (IPL). The core regions of DAN were located in the dorsal posterior parietal cortex and frontal eye fields. The CON was divided into two subnetworks (CON1, CON2) and consisted of regions in the insula, operculum, anterior cingulate cortex (ACC), and MFG. The frontopolar network included regions mainly in the frontopolar cortex. S1 Fig illustrates these networks in adults and children separately and shows that adults had two lFPNs (lFPN1, lFPN2), whereas children had only one lFPN. The spatial distribution of the core regions of the lFPN in children resembled that of the lFPN1 in adults. The lFPN2 that was not found in children was excluded from the subsequent analyses leaving in total 10 ICNs for further testing. In the following, we will describe group differences within and between the ICNs first during resting state and then during tasks. Thereafter we will delineate FC differences between resting state and task performance.

### Group differences in the FC of ICNs during resting state

During resting state, the between group comparison of the within-network FC (see section Functional MRI data analysis, Pipeline point 5) showed a significantly higher degree of co-activation in the ACC within the CON1 (peak voxel coordinates -6, 2, 36) (*p* < 0.05, FDR corrected, cluster size > 10 contiguous voxels) in children relative to adults (Fig 2a). No significant group differences were observed in the between-network FC.

### Group differences in the FC of ICNs during tasks

Using the 10 ICNs from the resting state (Fig 1) as spatial templates, the between group comparison of the FC during WM tasks (see section Functional MRI data analysis, Pipeline point 5) showed a significantly higher degree of connectivity within five ICNs in children relative to adults, representing the DMN, CON, and FPN (*p* < 0.05, FDR corrected, cluster size > 10 contiguous voxels) (Fig 2b). Table 1 lists the brain areas and peak voxel coordinates within the ICNs that showed significantly higher FC in children than adults. Since children compared to adults had lower task accuracy, we used the d’ as a covariate in the permutation tests to control for the group difference in the performance. After controlling for the task performance, the group differences in FC within the vDMN and CON1 remained significant with peak voxel coordinates in the retrosplenial cortex within the vDMN and in the ACC within the CON1 (Table 1). There were no brain regions where adults had stronger FC relative to children.

**Table 1:** The ICNs that showed stronger FC in children than adults in the task fMRI data.

ICN	Regions	Peak voxel			
		Vox	x	y	z
***ICNs of task-evoked FC***					
aDMN	**R medPFC/paraCG**: L medPFC/paraCG, SFG, L/R ACC, FP	831	18	38	24
vDMN	**L LG**:L/R LG, Cal, RC, preCun, PCC, R PHG, Cun	1108	-18	-62	0
rFPN	**R OFC**: SFGmed, CG, Sc, PCL, SMC	240	14	26	-12
CON1	**R ACC**: L ACC, L/R medPFC/paraCG, MFG, SFG, FP	1875	6	26	28
CON2	**L IFG**: Oper	94	-38	18	16
***ICNs of task-evoked FC with d’ as a covariate***					
vDMN	**R RC**: L Cal, RC, preCun	139	6	-58	8
CON1	**R ACC**	12	6	30	28

The MNI coordinates of the peak voxel and number of voxels in the voxel cluster are reported for each ICN expressing stronger FC in children compared to adults. The brain area corresponding to peak voxel is written in bold and anatomical areas included in the voxel cluster are written in regular. The data were FDR corrected at p < .05. ACC, anterior cingulate gyrus; Cal, calcarine cortex; CG, cingulate gyrus; Cun, cuneus; FP, frontal pole; IFG, inferior frontal gyrus; LG, lingual gyrus; MFG, middle frontal gyrus; OFC, orbital frontal cortex; Oper, operculum; paraCG, paracingulate gyrus; PCC, posterior cingulate cortex; PCL, paracentral lobule; PFC, prefrontal cortex; PHG, parahippocampal gyrus; preCun, precuneus; RC, retrosplenial cortex; Sc, subcallosal cortex; SFG, superior frontal gyrus; SMC, supplementary motor cortex; IC, independent component; med, medial part; Vox, number of voxels; L, left; R, right.

Full and partial correlations of FC strengths between networks and their hierarchical clustering during resting state and tasks (see section fMRI data analysis, Pipeline point 4) are illustrated in Fig 3a and 3b, respectively. Partial correlation analysis showed that adults relative to children had stronger FC between the aDMN and rFPN during tasks (Fig 3c). This group difference was caused by a significant positive correlation between aDMN and rFPN in adults but not in children. Children relative to adults had stronger FC between aDMN and the frontopolar network (Fig 3d), which resulted from a significant positive correlation between the networks in children and non-significant correlation in adults. After regressing out the effect of task performance, the stronger connectivity between the aDMN and the frontopolar network in children compared to adults remained significant, but the between-network connectivity between the aDMN and rFPN that was stronger in adults than children, disappeared.

### Group differences in FC strength of ICNs between resting state and tasks

In order to investigate group differences in FC between resting state and task performance, we used a two-way repeated measures ANOVA (see section fMRI data analysis, Pipeline point 6). The results showed significantly stronger mean FC within the rFPN in children than adults (main effect of group, *F*(1,28) = 8.5, *p* = 0.0069, partial *η2* = 0.23), and a significant group x brain state interaction (*F*(1,28) = 8.89, *p* = 0.0059, partial *η2* = 0.24), such that the mean FC within the rFPN was significantly stronger in children than adults during tasks (*t*(28) = 3.81, *p* = 0.0007) (Fig 4).

To evaluate whether the FC of the ICNs differed significantly between the brain states, we performed an additional voxelwise analysis for each ICN, separately for each group (see S3 File). In both groups, significantly stronger FC was observed during resting state than tasks in several ICNs, as illustrated in S2 Fig.

### Behavioral performance and correlations between age, behavior and FC

**Fig 5:**
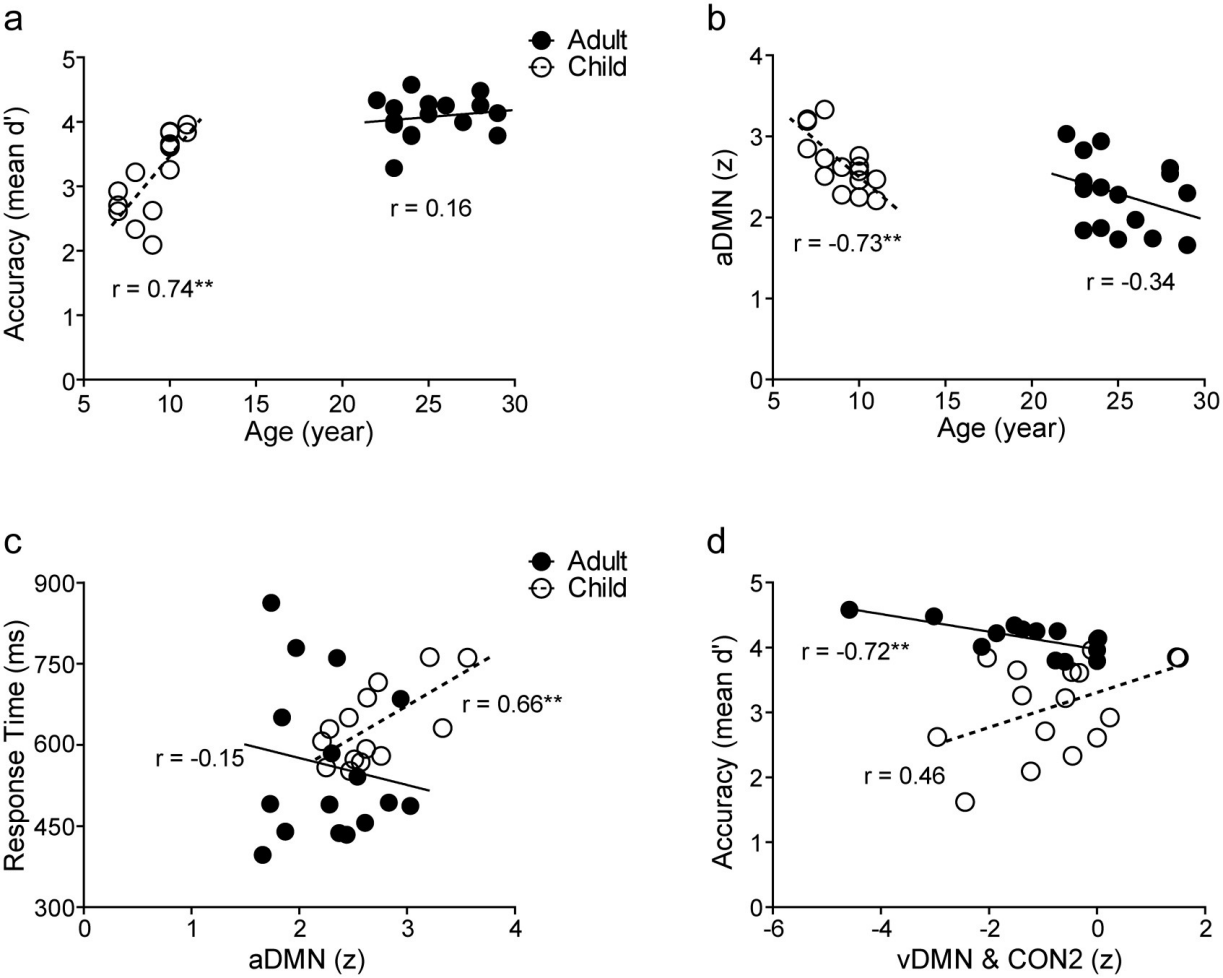
Correlations between age, behavior and FC during tasks. In children, accuracy of the task performance correlated positively with age (a), and the FC strength within the aDMN (the mean z-value of the aDMN network) correlated negatively with age (b) and positively with RT (c). In adults (d), the task performance accuracy correlated negatively with the between-network connectivity. CON, cingulo-opercular network; DMN, default mode network; a, anterior; v, ventral.

The two-way repeated-measures ANOVA with task (F, S, Fs, Sf) as a within-subjects factor and group (children, n = 16, adults, n = 16) as a between-subjects factor showed that adults performed the tasks with higher accuracy (d’; *F*(1,30) = 21.73, *p* < 0.0001, partial *η*^*2*^ = 0.42) than children but the RTs did not differ significantly between the groups (*F*(1,30) = 3.82, *p* = 0.06, partial *η*^*2*^ = 0.11). In children, accuracy of the task performance correlated positively with age (*r* = 0.74, *p* = 0.002) (Fig 5a). Table 2 shows the RT and d’ for all tasks in children and adults.

**Table 2:** The response times and accuracy (d’) in children and adults during tasks.

Performance	RT (ms) (Mean ± SD)		d’ (Mean ± SD)	
	Children	Adults	Children	Adults
F task	658.67 ± 104.43	572.06 ± 142.98	3.37 ± 0.83	4.17 ± 0.41
S task	680.42 ± 107.45	603.00 ± 144.95	3.38 ± 0.84	4.20 ± 0.42
Fs task	624.94 ± 103.21	529.47 ± 151.98	3.35 ± 0.86	4.32 ± 0.36
Sf task	615.86 ± 90.34	542.81 ± 139.82	2.83 ± 0.78	3.96 ± 0.47

RT, response time; F, face-1-back task; S, scene-1-back task; Fs, remember-face-ignore-scene task; Sf, remember-scene-ignore-face task.

In children, the FC strength within the aDMN was associated with age and RT. These correlations showed that the younger the children were, the stronger was the FC (*r* = -0.73, *p* = 0.002) (Fig 5b), and the stronger the FC, the slower the children responded (*r* = 0.66, *p* = 0.01) (Fig 5c). In adults, the task performance accuracy correlated with the between-network connectivity so that the more negative the correlation was between the vDMN and CON2, the better was the performance (*r* = -0.72, *p* = 0.004) (Fig 5d).

### Effects of motion on age, behavior and FC

No significant correlation was observed between any of the motion parameters (mean absolute displacement, maximum absolute displacement, mean relative displacement, maximum relative displacement) and age or any of the behavioral parameters (RT, number of hits, misses, FAs and performance accuracy (d’)) either in adults or children, before or after FIX cleaning.

We also calculated correlation analyses between motion residuals and FC. During resting state, there were no significant correlations between the motion residuals and FC either in adults or children.

During tasks, in adults, areas in the vDMN and CON1 showed significant correlation between the motion residuals and FC (p < 0.05, FDR corrected, cluster size > 10 contiguous voxels) (S3 Fig). In vDMN, the brain areas showing significant correlation included 50 voxels (out of 1108 voxels) in the left LG and precuneus/ retrosplenial cortex, and in CON1, 541 voxels (out of 1875 voxels) in the left and right ACC, paraCG, left MFG, SFG and frontal pole. In children, no areas showed significant correlations between the motion residuals and FC.

## Discussion

In the present study, we investigated resting state and task-evoked FC of neurocognitive ICNs in preadolescent children and young adults. We found that the intrinsic brain network architecture in 7-11-year-old children was comparable to that in young adults, but the FC strength during resting state and task performance and between the brain states differed between the groups.

In the following, we will first describe the ICN templates that were defined using the resting state fMRI data combined from the two groups. Then, we will discuss group differences in FC during resting state and task performance and between the brain states.

### ICN templates created from the combined group resting state fMRI data

The resting state network architecture has been suggested to represent an intrinsic, standard state of brain organization, and to be consistently present across multiple task states [17,21]. Thus, in the current study, we used the resting state ICNs as templates to investigate the group differences between children and adults of brain network FC within resting state and task performance and between the two states. In order to avoid statistical bias in group comparisons, we combined the resting state data from the two groups to produce the group ICA maps, and identified 11 neurocognitive networks as spatial templates for further analyses. We also used ICA for resting state fMRI data in each group separately and found that while adults had two lFPNs (lFPN1, lFPN2), children had only one lFPN, whose spatial distribution of core regions resembled that of the lFPN1 in adults. This result echoes the findings of de Bie et al. [37] that certain regions within the attention control networks identified in 5-8-year-old children appear in separate networks in adults. In the current study, we excluded from further analyses the ICN (lFPN2) that was found only in adults. We thus used in total 10 ICNs as spatial templates for the subsequent analyses to investigate the differences of brain network FC between children and adults. Overall, the results of the resting state ICA are in line with earlier literature [38,43,84] suggesting that the general architecture of ICNs is already established in young school aged children.

### Group differences in the FC of ICNs during resting state

During resting state, the direct between-group comparisons showed mainly similar FC of the ICNs in the two age groups. The finding of an adult-like network organization in 7-11-year-old children is in accordance with previous resting state fMRI studies using graph theory and showing that children older than seven years have similar resting state brain networks as adults with small-world clustering and average path lengths, suggesting that the connections between brain regions might already be capable of transmitting information efficiently [12,31,49]. Consistent with our hypothesis that although the architecture of the ICNs may already be adult-like in children, the network FC is still under fine-tuning, we found stronger resting state FC in 7-11-year-old children than in adults in the ACC within the CON1. This finding is in line with the results of recent developmental studies on resting state FC reporting stronger within-network connectivity in 10-12-year-old children than adolescents in CON [12], and a negative association between FC and age in 6-10-year-old children in ACC areas [85]. Together these results add to the current literature that suggests a non-linear development for neurocognitive networks with a positive association between FC and age in infancy [86] and early childhood [87], but a negative association with age from middle childhood till early adolescence [12,43,85]. In the current study, we did not find robust FC differences between children and adults during resting state. We used up-to-date methods for artifact removal, which largely removed the spurious results caused by excessive motion during scanning in children [84] that may have confounded some previously reported developmental differences in FC.

The between-group FC difference observed during resting state is likely related to the morphological developmental changes in the gray matter, such as synaptic pruning, that occur over many years up to young adulthood during brain maturation [88–89]. We therefore performed an additional voxel-based morphometry analysis to investigate possible differences between the groups in the grey matter volume (see S4 File). We found that the gray matter volume was significantly larger in children than adults in widespread cortical areas including the ACC within the CON1 (S4 Fig). This result lends support to the suggestion that the morphological developmental changes in the gray matter may partially explain the resting state FC differences between different age groups [43,90].

### Group differences in the FC of ICNs during tasks and between resting state and tasks

In the current study, participants performed visual 1-back WM tasks. WM refers to the ability to maintain and manipulate information in mind over a time period of several seconds [91]. The performance of WM tasks involves several cognitive processes such as attention allocation, memory maintenance and behavioral adjustments according to internal goals [92]. Consequently, WM activates brain areas of several neurocognitive networks. These areas include the dorsolateral- and ventrolateral PFC, frontopolar cortex, ACC, and posterior parietal cortex [93,94]. FC among WM-related brain areas plays an important role in the cognitive performance. For example, a recent study showed that increased FC strength among WM-related brain areas (PFC, posterior parietal cortex, and ACC), over a two-year follow-up period, was associated with improved WM performance in adults [95]. The findings of the current study also underscore the importance of functional network connectivity in WM by demonstrating age-dependent differences in FC: children, who as a group performed the tasks with less accuracy than adults, had stronger FC compared to adults within several WM-related networks (DMN, CON and FPN), stronger FC between the aDMN and frontopolar network, and weaker FC between the aDMN and rFPN. Moreover, the FC within the aDMN correlated with the age and response speed of the children: the younger the children were, the stronger the FC and the slower the response.

In the following, we will first focus on the group differences observed in our study regarding the DMN and the right FPN in and between the two brain states. When interpreting these findings, it is helpful to consider how FC results have been explained in earlier studies. First, it has been concluded that brain areas that are similarly modulated by tasks tend to exhibit correlated activity, even in the absence of tasks, and second that brain areas that show opposite responses during tasks tend to exhibit anti-correlated activity, i.e. the activity between the areas is negatively correlated [16,96]. Taking this into account, the group differences in the FC reported here may be best understood through findings of earlier fMRI studies showing that areas of the FPN increase their activity during cognitive task performance, and suggesting that the FPN is crucial for attention and WM [15–16,24,26, 28–29]. Accordingly, in our earlier study on 7-11-year-old children and young adults [60], using the same visual WM tasks as here, the 1-back tasks, in which subjects memorized face or scene images, activated predominantly the right sided frontoparietal areas in children and left sided areas in adults. These activation patterns may here be reflected in the stronger FC within the rFPN in children than adults during tasks (Fig 2b).

The DMN, in contrast, is involved in self-referential mental processes [22–25,97], and exhibits a consistent activity decrease during the performance of cognitively demanding tasks [16,27, 98–99]. In the current study, the aDMN was positively coupled with the rFPN in adults during tasks whereas this coupling was very weak in children (Fig 3c). The positive coupling between the networks in adults indicates that areas within the aDMN and rFPN changed their activity level in the same direction. As mentioned above, adults and children had different activation patterns during the tasks: adults activated the left frontoparietal areas and deactivated areas of the DMN more than children, whereas children relied more on the right frontoparietal areas [60]. These task-related activation patterns may partly explain the positive correlation between the aDMN and rFPN during task performance in adults resulting in deactivation of the DMN and downgrading of activation in the rFPN areas. In children, the coupling between these networks was weak which is also in line with their task-related activation pattern showing weaker deactivation of the DMN areas and activation of the rFPN areas.

Since children, compared to adults, had lower task performance accuracy, the observed group differences in FC during tasks might be associated with the level of task performance [100]. Therefore, we used the performance accuracy (d’) as a covariate in the group comparisons of FC. After controlling for the performance accuracy, three task-related group differences in FC remained significant suggesting that they were not related to the group difference in the performance of the tasks. They were the stronger FC in children than adults within the vDMN and CON1, and between the aDMN and the frontopolar network, whereas the group differences in FC within the aDMN, rFPN, and CON2, and between the aDMN and rFPN disappeared. This result further emphasizes that the DMN and FPN play an important role in WM task performance, and corroborates a recent study [101] showing that performance differences may be linked with the FC differences between the groups. However, the impact of motion on the FC of vDMN and CON1 cannot be totally excluded, since in adults, 4.5% of the voxels in vDMN, and 28.9% in CON1, showing within-network group differences, demonstrated significant correlations between motion residuals and FC.

We also investigated whether there were group differences in the FC of the ICNs between the resting state and task performance. This analysis showed an interaction between group and brain state such that children compared to adults modulated the FC strength regarding the rFPN differently between the brain states. In both groups, the within-network connectivity of the rFPN was positive during resting state and tasks, and during tasks, the FC was significantly stronger in children than adults. This finding shows that the two groups differed in the way how the within-network FC was adjusted during task performance compared to the resting state, and is in line with our hypothesis predicting that within-network connectivity would be stronger in children than in adults during tasks performance. The within-network FC differences between the groups were observed in task performance rather than in resting-state supporting the suggestion by Mennes et al. [102] that, instead of relying only on resting state data, the brain’s functional architecture should also be studied during the performance of different kinds of cognitive tasks.

### Associations of FC with age and performance

In children, age correlated with the FC strength of the aDMN and with task performance accuracy in such a way that the older the children were, the weaker was the FC within the aDMN during tasks and the better they performed the tasks. Moreover, the FC strength within the aDMN correlated also with the RT so that the weaker the FC within the aDMN, the faster the children performed the tasks. Together these relationships between age, network connectivity and performance demonstrate that the neurodevelopmental changes in the FC within the aDMN support better cognitive performance in preadolescent children. In line with this, the current study showed that during tasks, adults, who performed the tasks better than children, have weaker FC within the aDMN than children (Fig 2b). These findings lend support to recent neuroimaging studies in adults reporting that, cognitive task performance, compared to the resting state, decreases within-network FC [17], especially regarding the DMN [35], suggesting that a less modular brain network organization is related to facilitation of information processing during tasks [36].

Interestingly, the coupling between the vDMN and CON2 in adults was associated with task performance accuracy. This correlation was negative (Fig 5d), suggesting that the weaker the coupling between the networks was, the better was the performance. It is noteworthy, however, that in most adults, the coupling between the vDMN and CON2 was negative indicating anti-correlation between the networks. Thus, the more anti-correlated they were, the better was the performance. Regions of the DMN are involved in self-referential mental processes and usually deactivate during task execution [103] whereas areas of the CON are involved in attention, memory and executive functions and are activated during task performance [16,24]. The current result that links the coupling between these two networks with better performance in adults is in accordance with earlier studies showing that anti-correlations between task positive (areas that increase activation during task performance) and task negative networks (areas that decrease their activity during tasks) increase during development supporting better performance in cognitively demanding tasks [104–105].

### Advantages and limitations

An advantage of the present study is that the data were recorded during both resting state and tasks within the same imaging session in children and adults which allowed us to examine differences in the FC of the ICNs between resting state and task performance between the two age groups. Data-driven methods are currently popular in studies of human resting state networks, but are not commonplace in task-related functional imaging. Our study shows the applicability of data-driven approaches also to task-related functional imaging data analyses.

We are aware that subject motion is especially problematic in fMRI studies involving children who tend to move more than adults during scanning, which was also the case in the current study. Therefore, we applied FIX that takes into account and reduces the level of motion-related noise. After FIX cleaning, we also assessed the effect of motion residuals on group differences in the FC of the ICNs. As we show in the supplementary material (Table A-D in S5 File), the motion-related noise was significantly reduced for both age groups in the resting state and task-related datasets. Furthermore, within each group, the motion residuals during resting state did not significantly differ from those during tasks. In addition, there were no significant correlations between the motion residuals and within-network FC in children, suggesting that the between-group FC differences were not caused by movement of the children in the scanner.

The study has several limitations. The number of participants in the present study was relatively small, which may have decreased the power of our analyses to detect weak developmental effects and may limit the generalizability of our results. Despite this limitation, the current study detected the ICNs that are commonly reported in resting state studies. Another limitation is that it is still unsettled how the immediately preceding experience influences the spontaneous brain activity [72,98, 106–107]. In the current study, the subjects were first scanned during the WM tasks that could possibly have affected FC of the brain networks in the following resting state. The third limitation is that the data in the adult group were collected with two different scanners. However, the obtained group differences in the current study should not be caused by the scanner effect, since no significant FC differences were observed between the datasets from the two scanners (See S1 File).

In the current study, we compared the properties of the ICNs between two brain states, the resting state and the performance of visual WM tasks. In the future, the functional network properties should be studied across several types of tasks, e.g. by applying tasks with an increasing difficulty level or different tasks with equitable cognitive demands. Such an approach could provide better understanding of how FC varies between brain states.

## Conclusion

We investigated FC during both resting-state and task performance in 7-11-year-old children and young adults. Children had an adult-like pattern of the resting state ICNs, but the FC strength differed between the groups, especially during task performance. In general, during tasks, children compared to adults had stronger FC which was evident within the CON, DMN and rFPN, and between aDMN—frontopolar networks. Within the CON, children had stronger FC than adults also during resting state. When FC between the two brain states was compared, an interaction between group and brain state showed that within the rFPN, children had stronger FC during tasks than adults. When FC between the two brain states was compared, an interaction between group and brain state showed that within the rFPN, children had stronger FC during tasks than adults. Moreover, in children, the FC within the aDMN was associated with age and performance. These observations show that the ability to modulate the FC of the networks that support cognitive control and executive functions is still developing in 7-11-year-old children and suggest that mature FC is important for successful cognitive performance.

## Supporting information

S1 File Alertness of the participants during scanning and the comparison of FC data obtained using different scanners. (DOCX)

S2 File Classification of MELODIC ICA output of the resting state and task data. (DOCX)

S3 File Analysis of the FC differences between resting state and tasks within the groups. (DOCX)

S4 File Voxel-based morphometry analysis and results. (DOCX)

S5 File Assessing the motion correction effect. (DOCX)

S1 Fig Resting state networks in adults and children. (PDF)

S2 Fig Differences in FC strength of ICNs between resting state and tasks in children and adults. (PDF)

S3 Fig Significant correlation between motion residuals and FC in adults during tasks. (PDF)

S4 Fig Gray matter volume differences between children and adults. (PDF)
