# Supplementary material for: Functional connectivity of intrinsic cognitive networks during resting state and task performance in preadolescent children
Source: PLoS One. 2018 Oct 17;13(10):e0205690. doi: 10.1371/journal.pone.0205690 (PMC6192623; doi:10.1371/journal.pone.0205690)
Supplement: S1 File — (DOCX) [file pone.0205690.s001.docx]

**Supplementary results**

**Alertness of the participants during scanning**

After the imaging session, the participants rated the level of alertness during the first, middle and last parts of the scanning by using a 3-point scale (1= alert, 2=tired, 3= sleepy). A two-way repeated-measures ANOVA showed that there were no significant differences in the reported alertness during scanning between the groups (main effect of group: *F*(1,30) = 0.08, *p* = 0.785). Both groups reported that they were alert during the first part of the scanning, felt some tiredness in the middle compared to the first part (*t*(30) = 3.51, *p* < .0001, Cohen’s *d* = .54) and that their tiredness increased gradually towards the end of the session (the last part compared to the middle (*t*(30) = 5.91, *p* < .0001, Cohen’s *d* = .73) and first (*t*(30) = 5.71, *p* = .0014, Cohen’s *d* = 0.72) parts of the session).

**Comparison of FC data obtained using different scanners**

Eleven adults and all children of the present study were scanned with a 3 T General Electric (GE) Signa (Milwaukee, WI, USA) scanner and, after an update of the scanner, 5 adults were scanned with a 3 T Siemens MAGNETOM Skyra (Erlangen, Germany) scanner. To investigate whether there is a scanner effect on the results, we compared the within- and between-network FC between the data obtained with the two different scanners in adults. The within-network FC analysis was carried out using dual regression and permutation tests (5000 permutations) separately for the resting state and task data using the TFCE method [1] for voxel-wise multiple comparisons across the whole brain and the FDR correction [2] for multiple comparisons across the studied components. The resulting spatial maps were thresholded at p < 0.05 with cluster size > 10 contiguous voxels.

The comparison of between-network connectivity strengths was conducted separately for the resting state and WM task data by permutation tests (5000 permutations) with multiple comparison correction [3] using the FSLNets toolbox. No significant differences in FC strengths between the datasets obtained in the two different scanners were observed in the within- and between-network FC during resting state or tasks. The result suggests that the reported group differences in the current study should not be caused by a scanner effect.

The group comparisons and correlations reported in the main text are presented below in scatter plots with color coding for the adults indicating whether the Siemens or GE scanner was used.

**
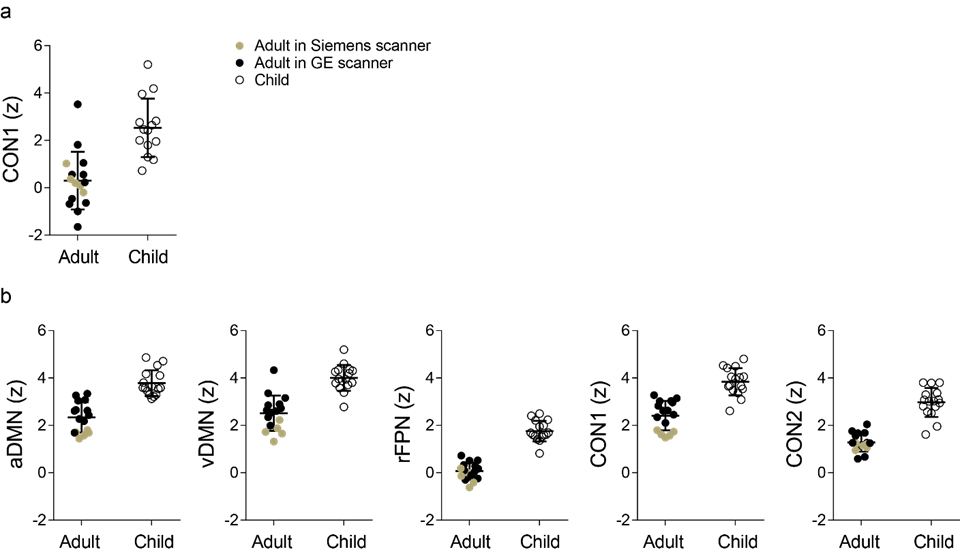
**

**Scatter plots of within-network FC of the ICNs during resting state and tasks.** The scatter plots illustrate the mean FC in each individual within the area that differed significantly between the groups (a) in resting state and (b) during tasks. The participants are color coded for the two scanners: 16 children (white circles) and 11 adults (black circles) were scanned with the GE scanner and five adults (gray circles) with the Siemens scanner. CON, cingulo-opercular network; DMN, default mode network; FPN, frontoparietal network; a, anterior; v, ventral; r, right.


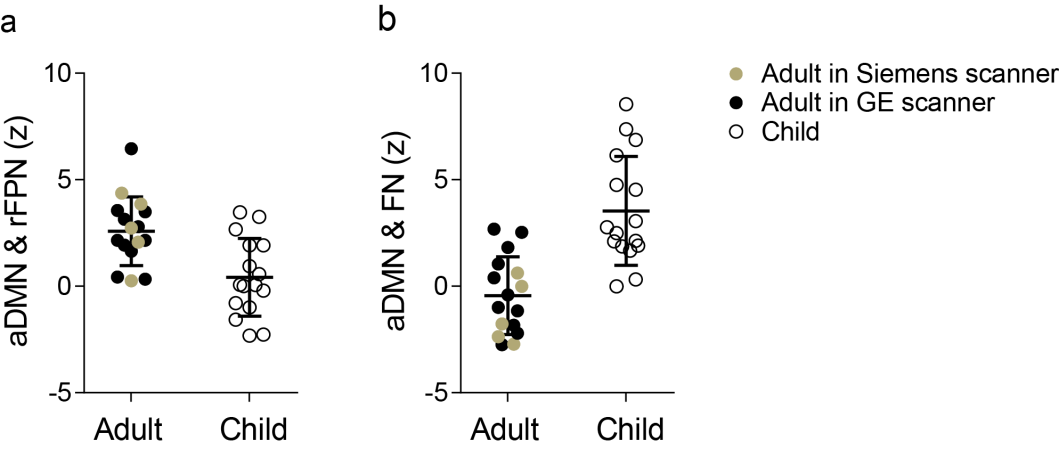


**Scatter plots of between-network FC of the ICNs during tasks.** The scatter plots illustrate the mean FC in each individual between the networks that differed significantly between the groups: (a) Between aDMN and rFPN (Adults > Children), (b) Between aDMN and FN (Children > Adults). The participants are color coded for the two scanners: 16 children (white circles) and 11 adults (black circles) were scanned with the GE scanner and five adults (gray circles) with the Siemens scanner. DMN, default mode network; FN, frontopolar network; FPN, frontoparietal network; a, anterior; r, right.


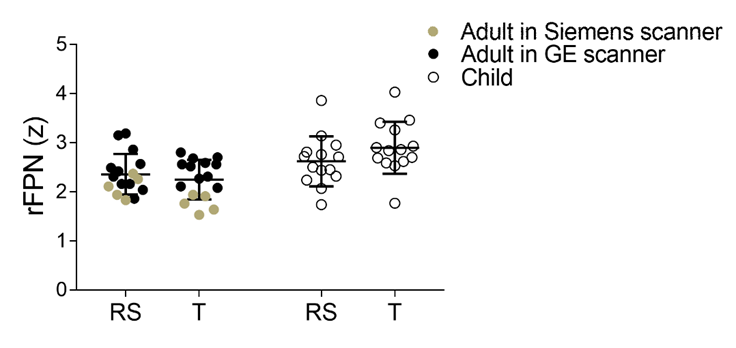


**Scatter plot of FC strength of the rFPN between resting state and tasks.** The scatter plot illustrates the average z-score across all voxels in the rFPN in each individual during resting state and tasks. The participants are color coded for the two scanners: 16 children (white circles) and 11 adults (black circles) were scanned with the GE scanner and five adults (gray circles) with the Siemens scanner. FPN, frontoparietal network; RS, resting state; T, tasks; r, right.


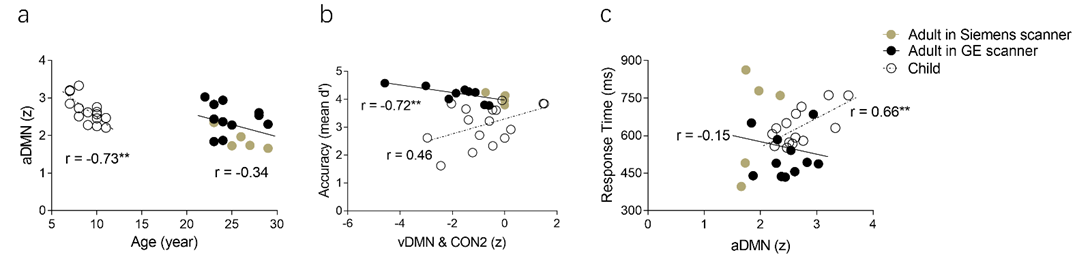


**Scatter plots of correlations between age, behavior and FC during tasks.** Scatter plots illustrate correlations between (a) the mean FC within the aDMN and age, (b) the mean FC between the vDMN and CON2, and performance accuracy, and (c) the mean FC within the aDMN and RT in each individual. The participants are color coded for the two scanners: 16 children (white circles) and 11 adults (black circles) were scanned with the GE scanner and five adults (gray circles) with the Siemens scanner. CON, cingulo-opercular network; DMN, default mode network; a, anterior; v, ventral.

**Supplementary references**

1. Smith SM, Nichols TE. Threshold-free cluster enhancement: addressing problems of smoothing, threshold dependence and localisation in cluster inference. NeuroImage. 2009; 44:83-98.

2. Benjamini Y, Hochberg Y. Controlling the false discovery rate - a practical and powerful approach to multiple testing. J R Stat Soc Series B. 1995; 57:289-300.

3. Smith SM, Beckmann CF, Andersson J, Auerbach EJ, Bijsterbosch J, Douaud G, et al. Resting-state fMRI in the Human Connectome Project. NeuroImage. 2013; 80:144-168.
