# Supplementary material for: Functional connectivity of intrinsic cognitive networks during resting state and task performance in preadolescent children
Source: PLoS One. 2018 Oct 17;13(10):e0205690. doi: 10.1371/journal.pone.0205690 (PMC6192623; doi:10.1371/journal.pone.0205690)
Supplement: S2 File — (DOCX) [file pone.0205690.s002.docx]

**Supplementary methods**

**Classification of MELODIC ICA output of the resting state and task data**

The FMRIB’s ICA-based Xnoiseifier – FIX (v1.061 beta) [1-2], which requires a supervised training sample to differentiate good and bad independent components (ICs) was used to regress out nuisance noises from the data. To create a training dataset for FIX, one of the authors (P.J.) visually inspected, classified and labeled the components into ‘good’ and ‘bad’ ones from a sample of our datasets. The sample datasets for hand-labeling were the resting state fMRI data from 14 adults and 14 children and task-related fMRI data from 16 adults and 16 children (one run of task fMRI data from each subject). Because resting state data were available altogether from 14 children, we included the same number of adults (n = 14) in the sample datasets of resting state data for training to avoid bias in different groups. The hand-labeled files were used to train the classifier and create the trained-weights files for resting state and task data separately. An IC was categorized as an artifact when it had such characteristics as: 1) low spatial overlap with gray matter or high spatial overlap with the sagittal sinus, white matter, and cerebrospinal fluid, or the brain’s boundary in structural templates, 2) a large number of small clusters, 3) predominantly high-frequency (> 0.1Hz) power in the time-course spectrum, and 4) the time series was bimodal or had sharp peaks or large jumps [2-3].

The leave-one-out (LOO) approach was used to evaluate the accuracy of the hand-classified data. The classification accuracy can be characterized in terms of two measures of success: ‘true positive rate’ (TPR, the percentage of true signal (‘good’) components correctly detected) and ‘true negative rate’ (TNR, the percentage of true artefact (‘bad’) components correctly detected). In the evaluation of the accuracy of the hand-classified data, a threshold was applied to determine the binary classiﬁcation of the components. Changing the threshold shifts the balance between the TPR and TNR; lowering of the threshold increases the TPR and decreases the TNR. The LOO test results showed that the mean TPR and TNR at a threshold of 20 that provided the best overall accuracy with the highest TPR in resting state and tasks, were 97.1% and 90.3% for the resting state data, and 97.0% and 90.4% for the task data.

Finally, the obtained trained-weights file was used to classify components in all the datasets. The bad components and motion confounds with 24 motion parameters were regressed out from the preprocessed 4D fMRI datasets.

**Supplementary references**

1. Griffanti L, Salimi-Khorshidi G, Beckmann CF, Auerbach EJ, Douaud G, Sexton CE, et al. ICA-based artefact removal and accelerated fMRI acquisition for improved resting state network imaging. NeuroImage. 2014; 95:232-247.

2. Salimi-Khorshidi G, Douaud G, Beckmann CF, Glasser MF, Griffanti L, Smith SM. Automatic denoising of functional MRI data: combining independent component analysis and hierarchical fusion of classifiers. NeuroImage. 2014; 90:449-468.

3. Smith SM, Beckmann CF, Andersson J, Auerbach EJ, Bijsterbosch J, Douaud G, et al. Resting-state fMRI in the Human Connectome Project. NeuroImage. 2013; 80:144-168.
