# Supplementary material for: Functional connectivity of intrinsic cognitive networks during resting state and task performance in preadolescent children
Source: PLoS One. 2018 Oct 17;13(10):e0205690. doi: 10.1371/journal.pone.0205690 (PMC6192623; doi:10.1371/journal.pone.0205690)
Supplement: S3 File — (DOCX) [file pone.0205690.s003.docx]

**Supplementary methods**

**Analysis of the FC differences between resting state and tasks within the groups**

We analyzed the differences of within-network FC between the resting state and task fMRI data in each group by using dual regression and permutation tests [1-2] that allow voxel-wise comparisons of FC patterns. The obtained 10 neurocognitive ICNs from the combined group ICA of the resting state data were used as templates for dual regression analysis. Dual regression was used to generate subject-specific versions of the spatial maps and associated time-series. First, the spatial maps of the templates were used as spatial regressors in a multiple regression analysis against the cleaned individual dataset. This resulted in a set of subject-specific time-series associated with each group-level spatial map. The individual time-series data were demeaned, variance-normalized, and then used as temporal regressors in a multiple regression analysis against the same dataset, resulting in a set of subject-specific spatial maps. These spatial maps were further transformed into maps with z-scores, which reflect the degree of within-network FC. Finally, we tested for statistically significant differences of within-network connectivity between resting state and tasks in each group using FSL's randomise nonparametric permutation-testing tool (5000 permutations) [3-4]. The threshold-free cluster enhancement (TFCE) method [5] was used to control for voxel-wise multiple comparisons across the whole brain and the FDR correction [6] was used to control for multiple comparisons across the studied components. The resulting spatial maps were thresholded at a *p*-level of 0.05.

**Supplementary references**

1. Beckmann CF, Mackay CE, Filippini N, Smith SM. Group comparison of resting-state FMRI data using multi-subject ICA and dual regression. OHBM. 2009

2. Filippini N, Machintosh BJ, Hough MG, Goodwin GM, Frisoni GB, Smith SM, et al. Distinct patterns of brain activity in young carriers of the APOE-epsilon4 allele. Proc Natl Acad Sci U S A. 2009; 106:7209-7214.

3. Nichols TE, Holmes AP. Nonparametric permutation tests for functional neuroimaging: a primer with examples. Human brain mapping. 2002; 15:1-25.

4. Winkler AM, Ridgway GR, Webster MA, Smith SM, Nichols TE. Permutation inference for the general linear model. NeuroImage. 2014; 92:381-397.

5. Smith SM, Nichols TE. Threshold-free cluster enhancement: addressing problems of smoothing, threshold dependence and localisation in cluster inference. NeuroImage. 2009; 44:83-98.

6. Benjamini Y, Hochberg Y. Controlling the false discovery rate - a practical and powerful approach to multiple testing. J R Stat Soc Series B. 1995; 57:289-300
