# Supplementary material for: Functional connectivity of intrinsic cognitive networks during resting state and task performance in preadolescent children
Source: PLoS One. 2018 Oct 17;13(10):e0205690. doi: 10.1371/journal.pone.0205690 (PMC6192623; doi:10.1371/journal.pone.0205690)
Supplement: S4 File — (DOCX) [file pone.0205690.s004.docx]

**Supplementary methods and results**

**Voxel-based morphometry analysis and results**

We applied an optimized voxel-based morphometry (VBM) protocol [1] using FSL tools [2] to assess group differences in gray matter volume. First, structural images of all the subjects were brain-extracted and gray matter-segmented before being registered to the MNI152 standard space using non-linear registration [3]. The resulting images were averaged and flipped along the x-axis to create a left-right symmetric, study-specific gray matter template. Second, all native gray matter images were non-linearly registered to this template and “modulated” to correct for local expansion (or contraction) due to the non-linear component of the spatial transformation: each voxel of each registered gray matter image was divided by the Jacobian of the warp field. The modulated gray matter images were then smoothed with an isotropic Gaussian kernel with a sigma of 2 mm (~5mm FWHM). Finally, voxel-wise GLM with permutation-based non-parametric testing (5000 permutations) and multiple comparisons correction across space using TFCE method was applied. A mask of whole-brain gray matter was used for the statistical testing of group differences in the whole-brain gray matter volume, and a mask of CON1 was used to test group differences in the gray matter volume within this network. A covariate was used for the statistical analysis to exclude the effect caused by different scanners. Gray matter voxels with corrected *p* < 0.05 were considered significant.

The results showed significantly larger gray matter volumes in children than adults in widespread cortical areas. Adults compared to children had larger gray matter volumes in restricted regions of the occipital cortex, cerebellum and subcortical regions (S4a Fig). We observed group differences in the gray matter volume in the brain regions that showed stronger FC in children than adults during resting state, i.e. the ACC within CON1 (S4b Fig).

**Supplementary references**

1. Good CD, Johnsrude IS, Ashburner J, Henson RN, Friston KJ, Frackowiak RS. A voxel-based morphometric study of ageing in 465 normal adult human brains. NeuroImage. 2001; 14:21-36.

2. Smith SM, Jenkinson M, Woolrich MW, Beckmann CF, Behrens TE, Johansen-Berg H, et al. Advances in functional and structural MR image analysis and implementation as FSL. NeuroImage. 2004; 23 Suppl 1:S208-219.

3. Andersson JLR, Jenkinson M, Smith S. Non-linear registration, aka spatial normalisation. Technical Report TR07JA2, Oxford Centre for Functional MRI of the Brain, 2007.
