# Supplementary material for: Functional connectivity of intrinsic cognitive networks during resting state and task performance in preadolescent children
Source: PLoS One. 2018 Oct 17;13(10):e0205690. doi: 10.1371/journal.pone.0205690 (PMC6192623; doi:10.1371/journal.pone.0205690)
Supplement: S5 File — (DOCX) [file pone.0205690.s005.docx]

**Supplementary results**

**Assessing the motion correction effect**

After standard preprocessing and using FIX to clean the data, the level of motion-related noise was significantly reduced in adults’ and children’s data during resting state and tasks. The statistical analyses were applied on values of motion parameters derived from MCFLIRT in FSL, including mean absolute displacement (Table A), maximum absolute displacement (Table B), mean relative displacement (Table C), and maximum relative displacement (Table D).

|  | Resting State | | | Tasks | | |
| --- | --- | --- | --- | --- | --- | --- |
|  | before FIX | after FIX | t-test | before FIX | after FIX | t-test |
| Child | 0.50±0.12 | 0.021±0.0025 | *t_(13)_* = 4.07,  *p* = .0013 | 0.55±0.11 | 0.021±0.0019 | *t_(15)_* = 4.92,  *p* = 0.00019 |
| Adult | 0.18±0.035 | 0.010±0.0012 | *t_(15)_* = 4.89,  *p* = 0.0002 | 0.15±0.017 | 0.010±0.0010 | *t_(15)_* = 8.75,  *p* < 0.0001 |
| G-Diff | *t_(15)_* = 2.56,  *p* = 0.022 | *t_(18)_* = 3.76,  *p* = 0.0014 |  | *t_(16)_* = 3.59,  *p* = 0.0025 | *t_(23)_* = 5.24,  *p* < 0.001 |  |

**Table A** Statistical analyses of the mean absolute displacement in children and adults

The values are averaged motion parameters of mean absolute displacement ± SEM (mm) in each group derived from MCFLIRT in FSL. The degree of freedom in the between group *t*-tests was corrected by Levene’s test for equality of variances. G-Diff, group difference

**Table B** Statistical analyses of the maximum absolute displacement in children and adults

|  | Resting State | | | Tasks | | |
| --- | --- | --- | --- | --- | --- | --- |
|  | before FIX | after FIX | t-test | before FIX | after FIX | t-test |
| Child | 2.30±0.60 | 0.056±0.0078 | *t_(13)_* = 3.77,  *p* = 0.0023 | 1.65±0.30 | 0.040±0.0035 | *t_(15)_* = 5.46,  *p* < 0.0001 |
| Adult | 0.44±0.081 | 0.028±0.0021 | *t_(15)_* = 5.18,  *p* = 0.00011 | 0.32±0.037 | 0.021±0.0013 | *t_(15)_* = 8.30,  *p* < 0.0001 |
| G-Diff | *t_(13)_* = 3.07,  *p* = 0.0086 | *t_(15)_* = 3.44,  *p* = 0.0037 |  | *t_(15)_* = 4.42,  *p* = 0.0086 | *t_(19)_* = 5.29,  *p* < 0.0001 |  |

The values are averaged motion parameters of maximum absolute displacement ± SEM (mm) in each group, derived from MCFLIRT in FSL. The degree of freedom in the between group *t*-tests was corrected by Levene’s test for equality of variances. G-Diff, group difference

|  | Resting State | | | Tasks | | |
| --- | --- | --- | --- | --- | --- | --- |
|  | before FIX | after FIX | t-test | before FIX | after FIX | t-test |
| Child | 0.14±0.029 | 0.019±0.0018 | *t_(13)_* = 4.36,  *p* = .00078 | 0.10±0.016 | 0.019±0.0011 | *t_(15)_* = 5.52,  *p* < 0.0001 |
| Adult | 0.045±0.0041 | 0.011±0.00066 | *t_(15)_* = 8.22,  *p* < .0001 | 0.036±0.0029 | 0.011±0.00074 | *t_(15)_* = 9.32,  *p* < 0.0001 |
| G-Diff | *t_(14)_* = 3.22,  *p* = 0.0064 | *t_(28)_* = 4.25,  *p* = 0.00021 |  | *t_(16)_* = 4.10,  *p* = 0.00085 | *t_(30)_* = 5.82,  *p* < 0.0001 |  |

**Table C** Statistical analyses of the mean relative displacement in children and adults

The values are averaged motion parameters of mean relative displacement ± SEM (mm) in each group, derived from MCFLIRT in FSL. The degree of freedom in the between group *t*-tests was corrected by Levene’s test for equality of variances. G-Diff, group difference

**Table D** Statistical analyses of the maximum relative displacement in children and adults

|  | Resting State | | | Tasks | | |
| --- | --- | --- | --- | --- | --- | --- |
|  | before FIX | after FIX | t-test | before FIX | after FIX | t-test |
| Child | 1.65±0.52 | 0.061±0.010 | *t_(13)_* = 3.05,  *p* = 0.0092 | 0.76±0.18 | 0.041±0.0039 | *t_(15)_* = 3.96,  *p* = 0.0013 |
| Adult | 0.17±0.029 | 0.030±0.0020 | *t_(15)_* = 4.76,  *p* = .00025 | 0.10±0.013 | 0.022±0.0012 | *t_(15)_* = 6.25,  *p* < 0.0001 |
| G-Diff | *t_(13)_* = 2.82,  *p* = 0.014 | *t_(14)_* = 2.93,  *p* = 0.011 |  | *t_(15)_* = 3.55,  *p* = 0.0029 | *t_(18)_* = 4.69,  *p* = 0.00019 |  |

The values are averaged motion parameters of maximum relative displacement ± SEM (mm) in each group, derived from MCFLIRT in FSL. The degree of freedom in the between group *t*-tests was corrected by Levene’s test for equality of variances. G-Diff, group difference
