# Supplementary material for: Functional connectivity of intrinsic cognitive networks during resting state and task performance in preadolescent children
Source: PLoS One. 2018 Oct 17;13(10):e0205690. doi: 10.1371/journal.pone.0205690 (PMC6192623; doi:10.1371/journal.pone.0205690)
Supplement: S1 Fig — (PDF) [file pone.0205690.s006.pdf]

S1 Fig. Resting state networks in adults and children

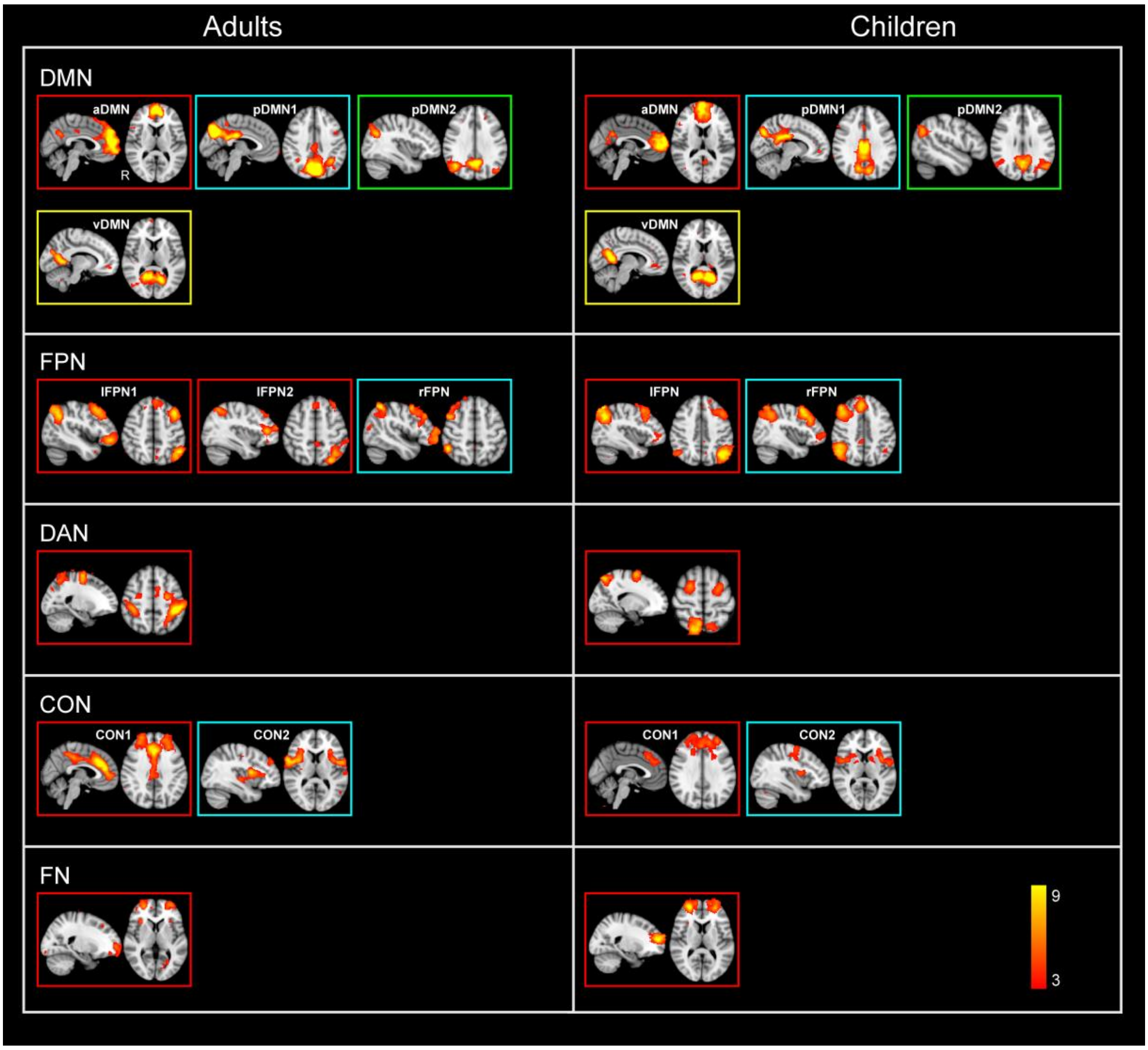

The resting state networks are illustrated in adults and children separately. The spatial maps of neurocognitive networks (thresholded at $Z > 3$) shown here represent anterior, posterior and ventral subnetworks of the default mode network (a/p/v DMN), and the left and right frontoparietal (l/r FPN), dorsal attentional (DAN), cingulo-opercular (CON), and frontopolar (FN) networks. The spatial maps are displayed in sagittal and axial views and superimposed on the MNI152 standard space template image. The adults had two lFPNs (lFPN1, lFPN2), whereas children had only one lFPN. The spatial distribution of the core regions of the lFPN in children resembled that of the lFPN1 in adults
