# Supplementary material for: Functional connectivity of intrinsic cognitive networks during resting state and task performance in preadolescent children
Source: PLoS One. 2018 Oct 17;13(10):e0205690. doi: 10.1371/journal.pone.0205690 (PMC6192623; doi:10.1371/journal.pone.0205690)
Supplement: S2 Fig — (PDF) [file pone.0205690.s007.pdf]

S2 Fig. Differences in FC strength of ICNs between resting state and tasks in children and adults

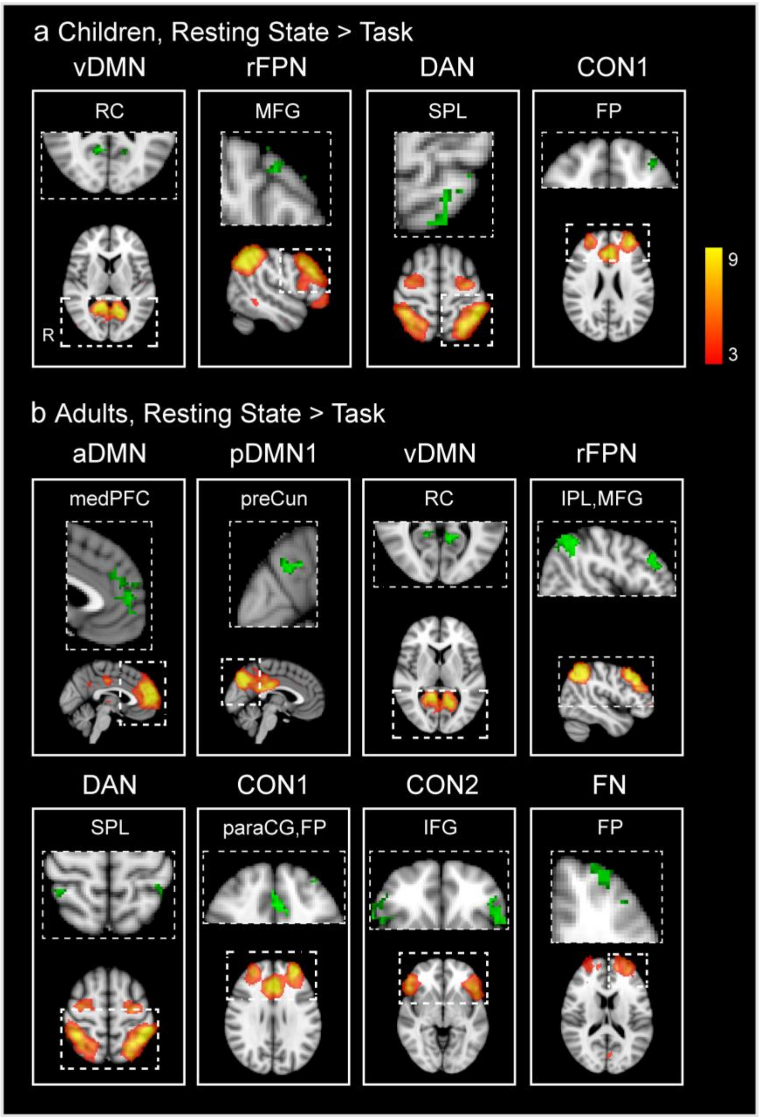

(a) In children, the FC strength within the vDMN, rFPN, DAN and CON1 was significantly reduced during tasks compared to resting state ($p < 0.05$, FDR corrected, cluster size > 10 voxels). (b) In adults, FC within eight ICNs that represented the three subnetworks of the DMN, the two CONs, the rFPN, the DAN and the FN ($p < 0.05$, FDR corrected, cluster size > 10 voxels) was significantly weaker during tasks compared to resting state. The statistical maps of significant FC differences are presented in green, and the corresponding group ICN templates are shown in red/yellow. All statistical maps are displayed on selected slice planes of the MNI152 standard brain template. CON, cingulo-opercular network; DAN, dorsal attentional network; DMN, default mode network; FN,

frontopolar network; FP, frontal pole; FPN, frontoparietal network; IFG, inferior frontal gyrus; IPL, inferior parietal lobule; MFG, middle frontal gyrus; paraCG, paracingulate gyrus; PFC, prefrontal cortex; preCun, precuneus; RC, retrosplenial cortex; SPL, superior parietal lobule; a, anterior; p, posterior; v, ventral; med, medial part; R, r, right.
