# Supplementary material for: Functional connectivity of intrinsic cognitive networks during resting state and task performance in preadolescent children
Source: PLoS One. 2018 Oct 17;13(10):e0205690. doi: 10.1371/journal.pone.0205690 (PMC6192623; doi:10.1371/journal.pone.0205690)
Supplement: S3 Fig — (PDF) [file pone.0205690.s008.pdf]

**S3 Fig. Significant correlation between motion residuals and FC in adults during tasks**

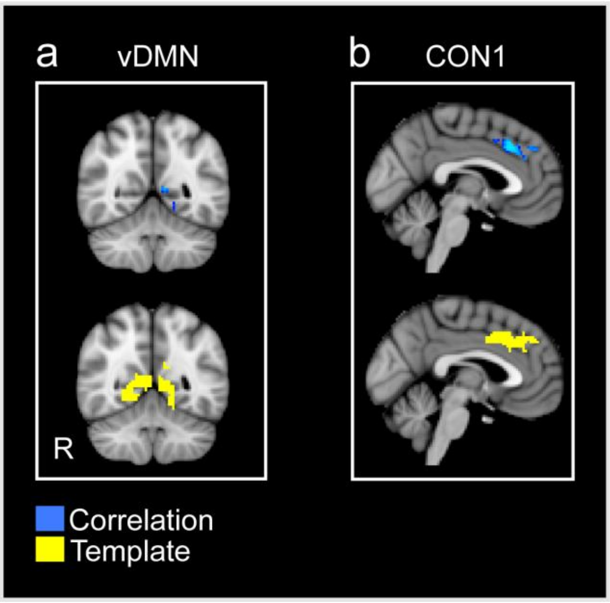


To evaluate the impact of motion residuals on the group differences of FC during resting state and tasks, we calculated the voxel-wise Pearson's correlation between the motion residuals and within-network FC metrics across participants, separately in adults and children [1]. The areas showing significant group differences of FC during resting state and tasks were used as masks for the correlation analyses. In children, there were no correlations between motion and FC during resting state or tasks. In adults, no areas showed significant correlations between motion residuals and FC during resting state. During tasks, voxels in the vDMN (a) and CON1 (b) showed significant correlation between motion residuals and FC ($p < 0.05$, FDR corrected, cluster size > 10 contiguous voxels). The voxels showing significant correlation were located in the left LG and precuneus/ retrosplenial cortex in the vDMN, and in the left and right ACC, paraCG, left MFG, SFG and frontal pole in the CON1.

## References:

1. Yan CG, Craddock RC, He Y, Milham MP. Addressing head motion dependencies for small-world topologies in functional connectomics. Front Hum Neurosci. 2013; 7:910.
