# Supplementary material for: Functional connectivity of intrinsic cognitive networks during resting state and task performance in preadolescent children
Source: PLoS One. 2018 Oct 17;13(10):e0205690. doi: 10.1371/journal.pone.0205690 (PMC6192623; doi:10.1371/journal.pone.0205690)
Supplement: S4 Fig — (PDF) [file pone.0205690.s009.pdf]

S4 Fig. Gray matter volume differences between children and adults

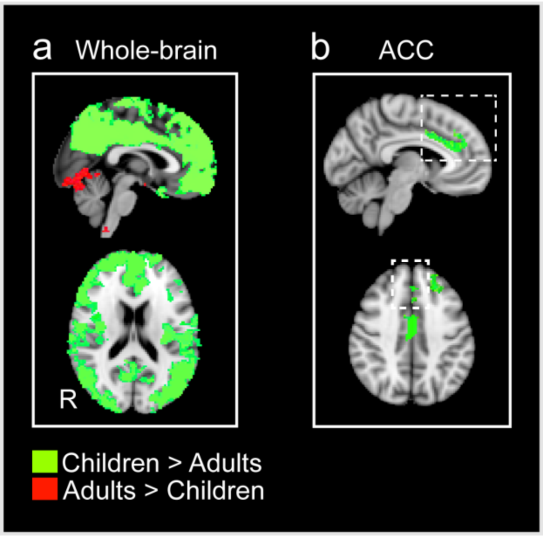

Group differences of the gray matter volume between children and adults were found (a) in widespread cortical areas and (b) in the brain regions that showed stronger FC in children than adults during resting state, i.e. in ACC within the CON1. All analyses were multiple comparison corrected at $p < 0.05$. The statistical maps of significant gray matter differences that were larger in children than adults are presented in green and those that were larger in adults than children in red. All statistical maps are displayed on selected slice planes of the MNI152 standard brain template. ACC, anterior cingulate cortex; R, right.
